# Thresholds for oximetry alarms and target range in the NICU: an observational assessment based on likely oxygen tension and maturity

**DOI:** 10.1186/s12887-020-02225-3

**Published:** 2020-06-27

**Authors:** Thomas E. Bachman, Narayan P. Iyer, Christopher J. L. Newth, Patrick A. Ross, Robinder G. Khemani

**Affiliations:** 1 Department of Biomedical Technology, Faculty of Biomedical Engineering, Czech Technical University in Prague, Kladno, Czech Republic; 2 Lake Arrowhead, USA; 3 Fetal and Neonatal Institute, Children’s Hospital Los Angeles, University of Southern California Keck School of Medicine, Los Angeles, CA USA; 4 Department of Anesthesiology and Critical Care Medicine, Children’s Hospital Los Angeles, University of Southern California Keck School of Medicine, Los Angeles, CA USA

**Keywords:** Pulse oximetry, Alarm fatigue, Neonatology

## Abstract

**Background:**

Continuous monitoring of SpO_2_ in the neonatal ICU is the standard of care. Changes in SpO_2_ exposure have been shown to markedly impact outcome, but limiting extreme episodes is an arduous task. Much more complicated than setting alarm policy, it is fraught with balancing alarm fatigue and compliance. Information on optimum strategies is limited.

**Methods:**

This is a retrospective observational study intended to describe the relative chance of normoxemia, and risks of hypoxemia and hyperoxemia at relevant SpO_2_ levels in the neonatal ICU. The data, paired SpO_2_-PaO_2_ and post-menstrual age, are from a single tertiary care unit. They reflect all infants receiving supplemental oxygen and mechanical ventilation during a 3-year period. The primary measures were the chance of normoxemia (PaO_2_ 50–80 mmHg), risks of severe hypoxemia (PaO_2_ ≤ 40 mmHg), and of severe hyperoxemia (PaO_2_ ≥ 100 mmHg) at relevant SpO_2_ levels.

**Results:**

Neonates were categorized by postmenstrual age: < 33 (*n* = 155), 33–36 (*n* = 192) and > 36 (*n* = 1031) weeks.

From these infants, 26,162 SpO_2_-PaO_2_ pairs were evaluated. The post-menstrual weeks (median and IQR) of the three groups were: 26 (24–28) *n* = 2603; 34 (33–35) *n* = 2501; and 38 (37–39) *n* = 21,058. The chance of normoxemia (65, 95%-CI 64–67%) was similar across the SpO_2_ range of 88–95%, and independent of PMA. The increasing risk of severe hypoxemia became marked at a SpO_2_ of 85% (25, 95%-CI 21–29%), and was independent of PMA. The risk of severe hyperoxemia was dependent on PMA. For infants < 33 weeks it was marked at 98% SpO_2_ (25, 95%-CI 18–33%), for infants 33–36 weeks at 97% SpO_2_ (24, 95%-CI 14–25%) and for those > 36 weeks at 96% SpO_2_ (20, 95%-CI 17–22%).

**Conclusions:**

The risk of hyperoxemia and hypoxemia increases exponentially as SpO_2_ moves towards extremes. Postmenstrual age influences the threshold at which the risk of hyperoxemia became pronounced, but not the thresholds of hypoxemia or normoxemia. The thresholds at which a marked change in the risk of hyperoxemia and hypoxemia occur can be used to guide the setting of alarm thresholds. Optimal management of neonatal oxygen saturation must take into account concerns of alarm fatigue, staffing levels, and FiO_2_ titration practices.

## Background

Shifts in SpO_2_ exposure have a profound impact on neonatal outcomes. Control of exposure is associated with the selection of a desired target range, selection of alarm limits as well as nursing compliance with good practices.

Manual titration of FiO_2_ to address unstable SpO_2_ is an arduous task. Infants in the NICU typically spend only about half the time in the desired range, and there is significant variation among centers [1]. Nursing intervention is driven by high and low SpO_2_ alarms, probably more than the prescribed target range. Oximeter alarms are notorious for false positives and are associated with alarm fatigue [2–4]. A persistent low alarm necessitates the need for increased supplemental oxygen to minimize the impact of transient hypoxemia, usually a result of respiratory instability. In contrast, high alarms usually signal the need to titrate the oxygen down following recovery from a marked desaturation. If the alarm limits are too narrow or the response to aggressive, troublesome swings between hypoxemia and hyperoxemia can occur. Further there is little evidence supporting guidelines and general practice with regard to selection of SpO_2_ alarm limits. Even consensus international guidelines for extremely preterm infants are not consistent. European Guidelines report there is weak evidence to support setting the alarms close to the desired target range [5]. Clearly doing so increases the frequency of false alarms and the potential for alarm fatigue [3, 6]. The most recent guidelines from the American Academy of Pediatrics, in contrast, suggest looser low alarms are more appropriate [7]. They further suggest that SpO_2_ alarm limits and target range should not only be decoupled, but also take into account the infant’s maturity. Neither guideline integrates the possible impact of differences in averaging period, alarm delay or differences in devices.

In the last two decades studies have focused on the intended SpO_2_ target ranges for the extremely premature with a resulting evolution of the standard of practice [1, 8]. The most recent very large studies suggest a higher, narrower target range might be preferred for extremely preterm infants [5, 9]. This perspective is, however, far from a consensus [8, 10–13]. Evaluations of the optimal SpO_2_ exposure for more mature infants are lacking. The risks associated with hypoxemia in near term infants are appreciated; however concerns about hyperoxemia have until recently been limited, at least compared to the extremely preterm.

We have developed an extensive SpO_2_-PaO_2_ database from our NICU and previously reported on the magnitude of the change of risk of severe hypoxemia and hyperoxemia across different SpO_2_ ranges [14]. The aim of this analysis was to see if specific SpO_2_ levels for selection of high and low alarms and target ranges could be identified based on the difference in the risk of hypoxemia and hyperoxemia and further to determine to what degree these thresholds might change depending on infant maturity.

## Methods

This is a prospectively defined analysis with the aim of describing arterial oxygenation levels (PaO_2_) associated with various possible SpO_2_ alarm limits and target ranges. The study is based on the paradigm that high and low SpO_2_ alarm limits should consider the risk of hypoxemia and hyperoxemia independent of the desired SpO_2_ target range and further consider infant maturity [7].

This study reflects infants in the Neonatal and Infant Critical Care Unit (NICCU) of Children’s Hospital Los Angeles. It is a tertiary care referral center affiliated with the Keck School of Medicine of the University of Southern California. The 58-bed NICCU receives transfers from the greater Southern California area. The bioethics review organization at Children’s Hospital Los Angeles (CHLA-17-00236) has waived the need for informed consent for aggregate data analysis studies and specifically approved this project.

In a previous publication we described the development of a SpO_2_-PaO_2_ database of infants receiving mechanical ventilator support with supplemental oxygen between August 2012 and July 2015 [14]. The database links arterial blood gas measurements in laboratory records with simultaneous SpO_2_ data from the patient monitor system. The SpO_2_ level is the mean of four 30-s readings coincident with the arterial sample. The gestational age from medical records for each infant, along with the date of measurement permitted calculation of post-menstrual age for each sample. The oximeter in the patient monitoring system used Masimo SET technology (Masimo Corporation Irvine, California), with 10 s averaging. Continuous monitoring of SpO_2_ is by practice post-ductal, pre-ductal assessments are conducted with another oximeter. Arterial samples were collected when clinically indicated. Umbilical catheters are used in most infants in their first week of life. As a matter of practice after that right radial lines are preferred, but when not possible left radial or posterior tibial lines are placed.

These study parameters were prospectively defined. Normoxemia was defined as PaO_2_ between 50 and 80 mmHg. Other oxemic levels were defined as severe hypoxemia (PaO_2_ ≤ 40 mmHg) and severe hyperoxemia (PaO_2_ ≥ 100 mmHg), We also evaluated levels below and above normoxemia (PaO_2_ < 50, > 80 mmHg). The selection of the severe thresholds was consistent with our previous publication. Also a consensus of the investigators, the potential ranges of SpO_2_ alarm limits were 85–89% and 95–98% and SpO_2_ target ranges within the envelop of 88–95%. The endpoints were the chance of normoxemia, and the risk of the 4 oxemic levels. Based on our previous work, we hypothesized that infant maturity would significantly impact the chance of normoxemia and risk of severe hyperoxemia and but not of severe hypoxemia. We used post-menstrual age (PMA) as the metric of maturity. PMA values were categorized into three groups. These were < 33 weeks, 33–36 weeks and > 36 weeks PMA. We felt that categories would be of more use clinically than a continuous effect. On a post hoc basis we also explored the impact of postnatal age.

Our primary measure was the risk or chance of each of these oxemic categories within the relevant SpO_2_ range. For the power analysis we assumed a baseline of relevant risk or chance of 25%, and considered sample sizes of PaO_2_ values for both 150 and 300 in an adjacent SpO_2_ bins. The range of 150–300 was selected as this was consistent with the numbers of observations in the smaller maturity categories at the SpO_2_ extremes. Based on this, we determined that there would be an 80% chance, at the *p* < 0.05 level, that we could detect a reduction to 12% with 150 observations and to 15% with 300 observations.

We treated each SpO_2_-PaO_2_ pair as an independent observation. We deemed consideration of within patient effects as not only impractical because of the large number of patients, but also inappropriate because of intra-patient sample variability of temperature, pH, PaCO_2_ and transfusion timing. Descriptive presentations of continuous data are shown as median and IQR, and of proportions as percent. The primary variables are presented as percentage along with their 95% confidence intervals of the proportion. Comparison of continuous variables used the Kruskal-Wallis test with Dunn’s procedure for pairwise comparisons. Comparisons of proportions were evaluated using the chi-square test, with Maracuilo’s procedure for pairwise comparisons. The impact of maturity on each of the three oxemic category parameters was tested by including maturity-category with SpO_2_, as independent variables, in a logistic regression equation with oxemic risk or chance as the dependent variable. For the exploratory analysis of the effect of postnatal age, we added age to this logistic regression model. A two-tailed *p* < 0.05 was considered statistically significant for all comparisons. Statistical tests were conducted with XLSTAT v19.02 (Addinsoft, Paris, France).

## Results

**Fig. 1:**
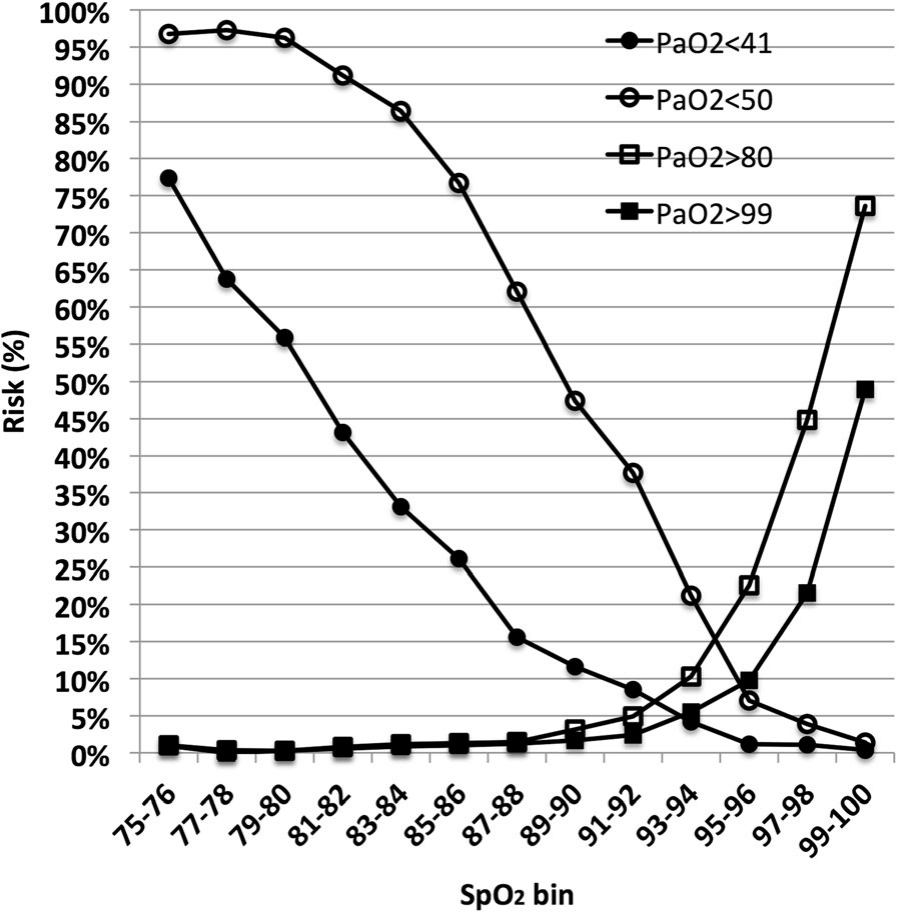
Risk of Hypoxemia and Hyperoxemia at different levels of SpO_2_. Circles represent hypoxemia (solid PaO_2_ < 41 mmHg, open < 50 mmHg). Squares represent hyperoxemia (solid PaO_2_ > 99 mmHg, open > 80 mmHg)

Our data included 26,162 SpO_2_-PaO_2_ observations of infants receiving supplemental oxygen and respiratory support over a 3-year period. Figure 1 provides a graphic overview of the risk of hypoxemia and hyperoxemia across SpO_2_ levels between 75 and 100%. The risk of each rises dramatically as SpO_2_ moves from a nominal target range. Even when moving within the latter the trade off between hypoxemia and hyperoxemia is obvious. It is also of note that the difference in risk of severe hypoxemia and a PaO_2_ < 50 mmHg, is much larger than the difference between severe hyperoxemia and a PaO_2_ > 80 mmHg.

For analysis these observations were divided into three groups according to post-menstrual age (PMA). Details characterizing the 3 groups are shown in Table 1. There were 2603 observations from 155 infants less than 33 weeks PMA, 2501 observations from 192 infants between 33 and 36 weeks PMA and 21,058 observations from 1031 infants greater than 36 weeks PMA. The number of observations per infant was similar among the three groups. The gestational age and post-menstrual age were consistent with the 3 maturity categories. The median SpO_2_ and PaO_2_ levels were lower in the group less than 33 weeks PMA. This group also included a higher share of measurements in normoxemia and less in severe hyperoxemia.

**Table 1:** Description of Maturity Category Cohorts

Maturity category	< 33 PMA	33–36 PMA	> 36 PMA	*p*
Subjects (n)	155	192	1031	na
Observations (n)	2603	2501	21,058	na
0bservations/subject (n)	12 (4–22)	9 (4–17)	11 (4–29)	< 0.01
GA (weeks)	26 (24–28)	34 (33–35)	38 (37–39)	< 0.001
PMA (weeks)	28 (26–31)	35 (34–36)	40 (39–43)	< 0.001
Postnatal age (weeks)	2.1 (1.0–3.7)	1.1 (0.4–2.3)	2.0 (1.0–6.3)	< 0.001
FiO_2_ (%)	45 (30–70)	50 (35–83)	45 (35–70)	< 0.001
SpO_2_ (%)	93 (86–97)	96 (91–100)	97 (87–100)	< 0.001
PaO_2_ (mmHg)	55 (45–71)	69 (50–100)	75 (47–112)	< 0.001
PaO_2_ ≤ 40 (%)	15%	12%	15%	< 0.001*
PaO_2_ 50–80 (%)	43%	33%	24%	< 0.001*
PaO_2_ ≥ 100 (%)	10%	25%	32%	< 0.001*
PaO_2_/FiO_2_	130 (81–192)	161 (90–240)	167 (93–263)	< 0.001
PaCO_2_ (mmHg)	45 (39–52)	45 (39–53)	45 (40–52)	ns
pH	7.34 (7.28–7.40)	7.36 (7.30–7.41)	7.39 (7.34–7.43)	< 0.001

Statistical comparisons (Kruskal-Wallis and chi-square* as appropriate) among the 3 maturity categories are shown in Table

The chance of normoxemia was dependent on SpO_2_ (*p* < 0.001) but not PMA. The chance of normoxemia across the range of 88–95% SpO_2_ was 65% (64–67 95% CI). The actual chance of normoxemia for 4 different overlapping SpO_2_ target ranges are shown in Table 2, and were different, specifically slightly lower in the lower ranges (*p* < 0.001). The PaO_2_ levels for each are also shown in the table and the differences between them are statistically significant (*p* < 0.001). Higher target ranges increase the possibility of higher levels of PaO_2_, but decrease the possibility of lower levels. The variation (interquartile range) of PaO_2_ levels among the 4 is similar.

**Table 2:** Chance of Normoxemia at Potential SpO_2_ Target Ranges

Target Range	88–92 SpO_2_	89–93 SpO_2_	90–94 SpO_2_	91–95 SpO_2_	*p*
n	2357	2946	3716	4584	
Chance 50–80 (%)	59% (57–61%)	63% (61–65%)	67% (65–68%)	68% (67–70%)	< 0.001
PaO_2_ (mmHg)	53 (47–61)	55 (48–64)	58 (51–68)	62 (53–73)	< 0.001

Normoxemia defined as PaO_2_ of 50–80 mmHg. Chance shown as a percentage (95% CI of proportion), differences evaluated with chi-square test. PaO_2_ levels show as median (IQR) differences evaluated with Kruskal-Wallis test

The risk of hypoxemia (PaO_2_ < 50 and < 41 mmHg) was independent of PMA but not SpO_2_ (p < 0.001). The risks at different potential alarm levels are shown in Table 3. The risks are not different at settings of 89, 88, and 87% SpO_2_ for either PaO_2_ < 50 mmHg or < 41 mmHg. They were both markedly higher at 86 and 85% SpO_2_. (*p* < 0.01) At these levels the risk of severe hypoxemia (< 41 mmHg) was marked; at 86% SpO_2_ (risk: 20% (16–24, 95% CI)) and at 85% SpO_2_ (risk: 25% (21–29, 95% CI)). The changes in risks are consistent with the changes in the PaO_2_ also shown in the table. The variation (interquartile range) of PaO_2_ levels is similar.

**Table 3:** Risk of Hypoxemia at Potential Low SpO_2_ Alarm Limits

	89% SpO_2_	88% SpO_2_	87% SpO_2_	86% SpO_2_	85% SpO_2_	*p*
n	279	251	331	389	444	
Risk < 50 (%)	46% (40–50%)	49% (40–55%)	50% (45–56%)	74% (70–78%)	71% (66–75%)	< 0.001
Risk < 41 (%)	13% (9–17%)	10% (06–14%)	11% (8–15%)	20% (16–24%)	25% (21–29%)	< 0.001
PaO_2_ (mmHg)	51 (44–57)	50 (44–54)	49 (44–55)	46 (42–50)	46 (40–50)	< 0.001

Severe hypoxemia defined as PaO_2_ of < 41 mmHg. Risks shown as a percentage (95% CI of proportion), differences evaluated with chi-square test. PaO_2_ among all levels presented as median (IQR), with differences in evaluated with Kruskal-Wallis test

The risk of hyperoxemia (PaO_2_ > 80 and > 99 mmHg) was significantly different among the 3 PMA categories (*p* < 0.001) and within each category among the SpO_2_ levels (*p* < 0.001). The actual risks at different potential alarm levels are shown in Table 4 for each maturity category. The potential point of marked increase in the risk of a PaO_2_ > 80 and > 99 mmHg were different for the three maturity categories. With regard to severe hyperoxemia, for those < 33 weeks it was a reading of 98% SpO_2_ (risk: 25% (18–33, 95% CI)), which was significantly higher than at 95 and 96% SpO_2_ (*p* < 0.05). It was a SpO_2_ reading of 97% for those 33–36 weeks (risk: 20% (14–25%, 95% CI)), which was not significantly higher than 95 and 96%. A reading of 96% for those > 36 weeks (20% risk: (17–22, 95% CI)), and the difference between all pairs was statistically significant (*p* < 0.001). A point of demarcation for the risks of PaO_2_ > 80 mmHg is 1 SpO2 level lower for each of the 3 PMA categories. The changes in risks are consistent with the changes in the PaO_2_ levels also shown in the table. The variation (interquartile range) of PaO_2_ levels is similar except at 98% SpO_2_, which is wider.

**Table 4:** Risk of Hyperoxemia at potential High SpO_2_ Alarm Limits by Maturity Category

	95% SpO_2_	96% SpO_2_	97% SpO_2_	98% SpO_2_	*p*
PMA < 33					
n	175	154	150	126	
Risk > 80 (%)	18% (12–23%)	12% (7–18%)	37% (30–45%)	45% (34–54%)	< 0.001
Risk > 99 (%)	7% (3–11%)	4% (1–7%)	14% (8–20%)	25% (18–33%)	< 0.001
PaO_2_	63 (54–73)	62 (54–73)	71 (60–88)	80 (63–100)	< 0.001
PMA 33–36					
n	156	172	190	225	
Risk > 80 (%)	28% (0.21–0.35)	26% (19–32%)	0.43 (36–50%)	0.61 (54–67%)	< 0.001
Risk > 99 (%)	10% (6–15%)	13% (8–18%)	20% (14–25%)	34% (28–40%)	< 0.001
PaO_2_	68 (61–81)	70 (62–81)	79 (67–92)	86 (73–108)	< 0.001
PMA > 36					
n	959	1156	1483	1729	
Risk > 80 (%)	28% (25–31%)	42% (39–45%)	56% (53–58%)	70% (68–72%)	< 0.001
Risk > 99 (%)	14% (10–14%)	20% (17–22%)	28% (26–30%)	42% (41–45%)	< 0.001
PaO_2_	70 (61–83)	76 (65–93)	84 (70–103)	94 (78–124)	< 0.001

Severe hyperoxemia defined as > 99 mmHg. Differences in risk evaluated with chi-square test. PaO_2_ presented as median (IQR) with differences evaluated with Kruskal-Wallis test. PaO_2_ pairs within each maturity category are also statistically different (p < 0.001) except the difference between 95 and 96% SpO_2_ in both the < 33 weeks and 33–36 weeks groups

Our exploratory analysis determined that postnatal age was an independent predictor of chance of normoxemia (*p* < 0.001) and risk of severe hyperoxemia (*p* < 0.001), but not severe hypoxemia. With increasing age the chance of normoxemia increased while the risk of hyperoxemia decreased. However the size of the effect predicted by the regression equation was quite small; that is changes of + 0.7% (normoxemia) and − 0.6% (severe hyperoxemia) for each week of age.

## Discussion

We evaluated a large database of neonatal SpO_2_-PaO_2_ observations paired with infant postmenstrual age. Our aim was to provide additional guidance to support the selection of SpO_2_ alarm levels and target ranges for neonates receiving supplemental oxygen. We identified a SpO_2_ range consistent with normoxemia, and showed how a target range could shift depending on a preference for avoiding higher or lower levels of PaO_2_. We showed that the risk of hyperoxemia and hypoxemia increases exponentially as SpO_2_ moves toward extremes. We found that the risk of severe hypoxemia does not become marked until a level well below common low alarm settings. Finally we found that the risk of severe hyperoxemia becomes marked at different levels depending on postmenstrual age and importantly at thresholds not consistent with standard practices. This report is, to our knowledge, the first to document these perspectives.

We evaluated four overlapping target ranges, each 4 wide with mid points of 90, 91, 92, and 93% SpO_2_. Our data showed that there was a similar chance of normoxemia across these potential target ranges, but slightly favoring the higher target ranges. This consistency also suggests that a wider target range, even 88–95% SpO_2_, would maintain a similar chance of normoxemia, but could be easier to maintain. A wider range at the low end has been suggested for extremely preterm infants [10, 11], in contrast to the European guidelines that recommend a higher target range [5]. Two recent reports of practices in Europe and the US reported that most target ranges were within this wider envelop, though more often narrower than seven but rarely 4 or less [1, 8].

Our analysis did not identify an effect related to maturity associated with normoxemia as we had expected. However our hypothesis was based on risk data of extreme PaO_2_ levels (< 41 and > 99 mmHg) at SpO_2_ levels between 90 and 95%, which is different from our normoxemia criteria (PaO_2_ 50–80 mmHg). Further the information about likely PaO_2_ values, consideration of which might align with maturity, ought to be useful in selecting a target range within these boundaries [11]. A clinical aversion to higher or lower PaO_2_ levels is reasonable. The consideration of a trade off of high and low oxygen exposure is supported by a landmark evaluation comparing the long term outcomes of nearly 5000 extremely preterm infants randomized to one of two SpO_2_ target ranges (85–89% or 91–95%) [9]. It found the high range was associated with increases in severe retinopathy of prematurity and more likely need for supplemental oxygen at 36 weeks PMA, but lower levels of necrotizing enterocolitis and death.

Alarm fatigue in the NICU is a serious problem. Pulse oximetry, while an essential tool, generates the most false alarms and is the alarm least likely to be associated with an actionable nursing intervention [2, 3, 15]. It is not uncommon with unstable infants to experience a SpO_2_ alarm every few minutes, while an intervention is often only warranted every 5–10 min. Faced with this dilemma nurses have been shown to disregard alarm policy [1]. Attention to selection of reasonable alarm settings (delay, and level) as well as sensor/probe integrity, can impact the frequency of alarms not needing intervention [16, 17]. However setting alarms, whether by policy or practice, to avoid excessive frequency must also consider the risk of missing or delaying response to important events. Policy and practice must balance the need to find an acceptable medium to balance the risks associated with each. Our data provide SpO_2_ thresholds that are associated with marked hyperoxemia and hypoxemia. It is reasonable to consider a buffer zone between the alarm setting and the level of SpO_2_ concern. In addition, many events are short and it is standard practice to set the alarm delay to avoid these transient events not needing intervention. Correspondingly it seems appropriate to set a longer alarm delay when the buffer zone is wider.

Our data indicate that the risk of hypoxemia is not related to maturity and is not marked until the SpO_2_ is at 86% or 85%, at which point the risk is increasing exponentially. In contrast we found no relevant difference in risk at levels between 87 and 89%. Setting the low alarm between 87 and 89% SpO_2_ would create a buffer but at the expense of increased false alarms and alarm fatigue, without a compensating longer alarm delay. A recent analysis has determined that episodes that are significantly lower (< 80% SpO_2_) and prolonged (> 60 s) are related to bad outcomes [18]. However, we speculate that episodes of SpO_2_ with a nadir between 87 and 89% even if prolonged, would not have a clinical impact, because of the low risk of severe hypoxemia. Finally, based on an audit of extremely preterm infants in 83 NICUs, Hagadorn et al. reported good compliance with low SpO_2_ alarm unit guidelines, but provided no related details on the actual settings [1].

In preterm infants we found the risk of hyperoxemia did not become marked until SpO_2_ reached 97–98% in those < 33 weeks PMA and those 33–36 weeks PMA. This is higher than the most recent recommendations for setting the high SpO_2_ alarm around 95% in extremely preterm infants [5, 7, 10]. Such a lower setting could be appropriate with two difference rationales. It could be considered an appropriate buffer zone. But it certainly would increase false positive alarms, without a compensating longer alarm delay. It might also be appropriate if the goal was to avoid PaO_2_ levels approaching 80 mmHg, in alignment with a lower target range. Consistent with this likely excessive false positive rate from tighter high alarms, Hagadorn reported only 63% compliance with high SpO_2_ alarm unit guidelines [1].

In contrast to preterm infants, we found that the risk of hyperoxemia, PaO_2_ > 80 and > 99 mmHg, in infants > 36 weeks PMA was marked at a SpO_2_ of 96%. While reports of guidelines are sparse [19, 20], it is our impression that upper alarms for near term populations are often set much higher than 96%. This practice provides no buffer zone and certainly increases false negatives that could increase clinical risk of hyperoxemia. The concern about the risks associated with hyperoxemia in near term infants is less prevalent than in preterms. Nevertheless, hyperoxemia in children and adults has been associated with morbidity and mortality [21, 22] and it is reasonable to project these risks to near term infants.

The shift of the oxy-hemoglobin dissociation curve with increasing maturity that one would anticipate, was evident in high levels of SpO_2_ but not at moderate and low levels. While the predicted shift in the SaO_2_-PaO_2_ relationship is characterized in a shift of P50, it is understandable that the smaller predicted shifts in SpO_2_ at lower levels would be muted. The lack of precision and bias of the pulse oximeter, especially in these ranges, as well as other factors such as local perfusion are documented [23]. The transition from fetal to adult hemoglobin is quite predicable over a couple months of life in healthy neonates, but we did not identify a meaningful impact associated with postnatal age. However the transition from fetal hemoglobin is affected by treatment and disease severity. Transfusions have a marked effect [24–26]. Our study population, all transferred for a higher level of care, commonly were transfused. Accordingly, transfusion naive infants would be shifted more to the left [14]. Such a shift would reduce the risk of hyperoxemia.

This study’s design has several limitations. First the PaO_2_ thresholds we used for hypoxemia, normoxemia and hyperoxemia, while generally accepted, have not been validated with regard to outcome risk. It is unlikely they ever will be. There is a need for and a growing body of data correlating SpO_2_ exposure and outcomes. Of particular interest is a pending analysis of the impact of the actual, rather than assigned, SpO_2_ exposure in the NeOProM population [9]. We speculate that these interpretations will be easier with a better understanding of the relationship between PaO_2_ and SpO_2_. Other factors such as small for gestational age and hemoglobin level as well as cerebral and intestinal oxygenation are also relevant. Second, the study is observational. The location of the SpO_2_ sensor and site of arterial sampling were not controlled. It is likely that some of the paired comparisons do not reflect pre-ductal assessment. This could increase the variance, but we do not think this would have a relevant effect on the bias of the risk (median values). Third, we categorized the hyperoxemic risk into three PMA groups. These are reasonable groupings, but it is probable that the effect is somewhat continuous with increasing maturity, but certainly not strictly categorical.

Whether using these results to design research or to evaluate unit guidelines, several generalizability issues should be considered. The first is comparabilty to our study population. Our unit is referral based, with all infants transferred in for tertiary care. After intervention and recovery infants are often returned when they only need low levels of inspired oxygen and minimal pressure support. As reported their supplemental oxygen requirements are quite high. Also previously noted, as a result of transfusions, their oxy-hemoglobin relationship is shifted to right. Illustrative of this, in our least mature cohort we identified an incidence of severe hyperoxemia more than 10 times higher than that reported in a more traditional inborn population during the first week of life [27]. Another important consideration is the averaging and alarm delay settings on the oximeter. One large study confirmed the clinical relevance of these settings [28]. They documented a marked decrease in the incidence of severe hypoxemic events with increasing averaging time, and also demonstrated that it was associated with increased duration of episodes. They recommended using shorter averaging times and longer delays. Finally the oximeter measurement itself must be considered. Our data reflect a good bit of scatter in the PaO_2_ at each SpO_2_ level. Sources of the scatter seen with SpO_2_ monitoring are well described [13, 29]. .Consideration of differences in oximeter brands, and models should be considered as well. Our group previously reported no difference in bias between the Massimo and Nellcor devices across the range of saturations in the PICU, but did identify a problem with the use of inappropriate sensors [23]. Of more potential relevance, a difference between the Massimo and Nellcor oximeters has been reported in the SpO_2_ range of 87–90% [30]. While this difference is within the device’s 3% accuracy specifications, it might well effect a decision about selecting a lower target range, or the low SpO_2_ alarm setting.

## Conclusion

We provide quantification of the rate at which the risk of hyperoxemia and hypoxemia increase exponentially as SpO_2_ moves towards extremes, and how it is affected by maturity. Postmenstrual age influences the threshold at which the risk of hyperoxemia became pronounced, but PMA did not alter the threshold for hypoxemia or normoxemia. The thresholds at which a marked change in the risk of hyperoxemia and hypoxemia occur can be used to guide the setting of alarm thresholds. These findings support reconsideration of common alarm treshold practices. In extreme preterm infants, but not in more mature infants, high SpO_2_ alarms may be set higher than 96%. Likewise low SpO_2_ alarms may be set lower than 89%. SpO_2_ targeting ranges may be selected within the range of 88–95% SpO_2_. Optimal management of neonatal oxygen saturation must take into account concerns of alarm fatigue, staffing levels, and FiO_2_ titration practices. Integration of these factors should be evaluated in quality improvement programs.

## Data Availability

The data sets generated and analyzed during this study are not currently publically available, but are available from the corresponding author on reasonable request.

## Abbreviations

FiO_2_: Fraction of inspired oxygen
SpO_2_: Arterial oxygen saturation measured noninvasively
NICU: Neonatal intensive care unit
PaO_2_: Arterial partial pressure of oxygen (mmHg)
PaCO_2_: Arterial partial pressure of carbon dioxide (mmHg)
PMA: Post-menstrual age (weeks)
